# Dulaglutide‐combined basal plus correction insulin therapy contributes to ideal glycemic control in non‐critical hospitalized patients

**DOI:** 10.1111/jdi.13093

**Published:** 2019-06-28

**Authors:** Nobutoshi Fushimi, Takashi Shibuya, Yohei Yoshida, Shun Ito, Hiroki Hachiya, Akihiro Mori

**Affiliations:** ^1^ Department of Endocrinology and Diabetes Ichinomiyanishi Hospital Aichi Japan

**Keywords:** Glucagon‐like peptide‐1 receptor agonist, Inpatients, Type 2 diabetes

## Abstract

**Aims/Introduction:**

We investigated whether dulaglutide (DU)‐combined conventional insulin therapy is beneficial for glycemic control in non‐critically ill hospitalized patients with type 2 diabetes.

**Materials and Methods:**

This study was a prospective, randomized controlled pilot study. Participants were randomized to either basal‐plus (BP) therapy, where basal insulin and corrective doses of regular insulin were administered before meals, or BP + DU therapy, where BP therapy was combined with DU. Blood glucose (BG) levels before and after every meal were measured for 7 days after assignment to groups. Because we consider the ideal BG during hospitalization to be within 100–180 mg/dL, we defined this range as the hospitalized ideal glucose range (hIGR). We compared the percentage of BG measurements within the hIGR among all BG measurements (%hIGR), mean BG, glucose variability and insulin dose between the two groups.

**Results:**

Of 54 patients, 27 were assigned to the BP group and 27 to the BP + DU group. The %hIGR was significantly higher (44% vs 56%, *P* < 0.001), and the frequency of BG >240 mg/dL and BG <70 mg/dL was significantly lower in the BP + DU group than in the BP group (both *P* < 0.001). The mean BG (183 ± 29 vs 162 ± 30 mg/dL,* P* < 0.05), standard deviation (*P* < 0.01), coefficient of variation (*P* < 0.01) and total regular insulin dose (*P* < 0.05) in the BP + DU group were significantly lower than those in the BP group. No significant side‐effects were observed in either group.

**Conclusions:**

BP + DU therapy reduced the frequency of hyperglycemia and hypoglycemia, and resulted in a lower glucose variability.

## Graphical abstract

Dulaglutide‐combined insulin therapy contributes to ideal glycemic control in non‐critically ill hospitalized patients. This suggests the possibility of using a glucagon‐like peptide‐1 receptor agonist in the control of blood glucose in hospitalized patients, which is considered a new proposal for blood glucose control method for hospitalized patients

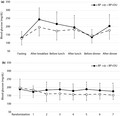

## Introduction

Numerous observational studies have shown that hyperglycemia increases poor clinical outcomes^1^, ^2^, ^3^, ^4^, ^5^, and treatment of hyperglycemia is associated with decreased mortality and morbidity among hospitalized patients^6^, ^7^, ^8^. However, hypoglycemia in hospitalized patients also increases mortality and morbidity^9^, ^10^, ^11^. Therefore, guidelines recommend avoiding iatrogenic hypoglycemia^12^, and thus, hospitalized patients with diabetes require glycemic control with low glycemic variability to prevent both hyperglycemia and hypoglycemia. Major guidelines recommend a narrow target blood glucose level of <180 mg/dL without hypoglycemia in non‐critical hospitalized patients^12^, ^13^ Glycemic variability (GV) in hospitalized patients also prolongs hospital stay and increases mortality^14^, ^15^, ^16^. In most instances in hospital settings, insulin is the preferred treatment for glycemic control, and it is recommended that most critically ill inpatients receive insulin infusion therapy, and non‐critical inpatients receive basal–bolus insulin therapy (BBT)^12^. However, the basal–bolus regimen is associated with a high risk of hypoglycemia.

Hypoglycemia has been reported in 12–32% of patients with type 2 diabetes in general medicine and surgery who were treated with the basal–bolus insulin regimen^17^. The use of oral antidiabetic drugs is generally not recommended for patients admitted to the hospital because of a paucity of data on their safety and efficacy. As such, the safety and efficacy of non‐insulin antihyperglycemic therapies in hospital settings are areas of active research^12^, ^13^.

Incretin agents are associated with a lower risk of hypoglycemia and less GV owing to their glucose‐sensitive mechanisms of insulin release and glucagon suppression^18^, ^19^. The incretin agent, glucagon‐like peptide‐1 (GLP‐1) receptor agonist (GLP‐1RA), is injected and can therefore be administered even in patients with difficulty in oral intake. The antihyperglycemic effect of GLP‐1RA is stronger than that of dipeptidyl peptidase‐4 inhibitors belonging to the same family of drugs^20^. This suggests that combining a GLP‐1RA with conventional insulin therapy might prevent both hyperglycemia and hypoglycemia, and thus achieve more ideal glycemic control. In the present study, we compared the GLP‐1RA, dulaglutide (DU), in combination with conventional insulin therapy and conventional insulin therapy alone, in non‐critical hospitalized patients.

## Methods

The present study was a prospective, randomized, open‐label, single‐center, controlled pilot study. Patients aged >18 years with type 2 diabetes and a known history of >3 months with type 2 diabetes, and who were treated at home with either diet alone, any combination of oral antidiabetic agents or low‐dose insulin therapy at a daily dose of <10 units/day before admission were eligible for participation in the study. Patients were enrolled into the study after admission to the hospital for a non‐critical illness with medical or surgical consequences. Exclusion criteria included admission to the intensive care unit (ICU), a history of diabetic ketoacidosis or hyperosmolar state, blood glucose (BG) >400 mg/dL before admission to the hospital, systemic steroid use, pregnancy, a history of pancreatitis or active gallbladder disease, impaired renal function with estimated glomerular filtration rate (eGFR) <30 mL/min per 1.73 m^2^, tube feeding, hypersensitivity to DU or inability to provide informed consent.

On admission, use of oral antidiabetic agents was discontinued, and patients were randomly assigned to either basal plus (BP) therapy, receiving basal insulin (glargine 100 U/mL; Eli Lilly and Company, Indianapolis, IN) once daily and corrective doses of regular insulin (Humulin R; Eli Lilly and Company) before meals, or to BP + DU therapy, receiving the BP regimen combined with DU (Trulicity; Eli Lilly and Company Indianapolis, IN, USA). The study started 7 days after patient assignment. BG was measured before every meal and 2 h after meals (or every 6 h if a patient was not eating). In addition, BG was measured at any time if a patient experienced symptoms of hypoglycemia or if requested by the treating physician. Glutest neo alfa^®^ (Sanwa Kagaku Kenkyusho, Nagoya, Japan) was used as the glucose meter.

Patients in both groups received a starting daily dose of glargine of 0.25 units/kg/day, except for those patients aged >70 years and/or with eGFR <50 mL/min per 1.73 m^2^, who received glargine at a starting daily dose of 0.15 units/kg/day. The dosage of DU injected on the first day of the study was 0.75 mg. The goal of therapy was to maintain a fasting BG (FBG) concentration of 100–140 mg/dL by daily adjustment of basal insulin according to protocol. If FBG was between 100 and 140 mg/dL on the previous day, the dose of glargine was maintained. If FBG was between 140 and 180 mg/dL on the previous day, the dose of glargine was increased by 10% every day. If FBG was >180 mg/dL on the previous day, the dose of glargine was increased by 20% every day. If FBG was between 70 and 99 mg/dL, the dose of glargine was decreased by 10% every day. If FBG was <70 mg/dL, the dose of glargine was decreased by 20% every day^7^, ^21^, ^22^, ^23^. The correctional sliding scale for insulin dosage was used to determine the appropriate insulin dose based on sensitivity to insulin (Table 1). Patients who were considered sensitive to insulin treatment (eGFR <50 mL/min per 1.73 m^2^, age >70, body mass index <20 kg/m^2^) were identified as “sensitive,” those potentially resistant to insulin (body mass index >30 kg/m^2^) were identified as “resistant” and the rest were categorized as “usual.”

**Table 1:** Supplemental insulin scale

BG (mg/dL)	Regular insulin (units)		
	Sensitive	Usual	Resistant
141–180	0	2	4
181–220	2	4	6
221–260	4	6	8
261–300	6	8	10
301–350	8	10	12
351–400	10	12	14
>401	12	14	16

The numbers in each column indicate the number of units of regular insulin. BG, blood glucose.

Hypoglycemia was defined as an episode of symptomatic hypoglycemia or BG >70 mg/dL. Hypoglycemic patients who were able to swallow received a 10‐g pure glucose tablet orally, and patients who were unable to take glucose orally received the equivalent of 10 g glucose by intravenous infusions of 50% glucose.

Treatment failure was arbitrarily defined as an average daily BG >300 mg/dL or two consecutive values of BG of >240 mg/dL before meals. If treatment failure occurred, patients in either group were switched to BBT or intravenous insulin infusion therapy.

We defined the ideal glucose range (hIGR) for hospitalized patients as within 100–180 mg/dL, and defined the primary outcome (%hIGR) as the percentage of BG measurements within the hIGR among all BG measurements (%hIGR: number of BG measurements within hIGR / all BG measurements × 100). The secondary outcomes were individual mean BG, GV (standard deviation and coefficient of variation), BG at evaluation time in each group, frequency of hypoglycemia, insulin dose and frequency of gastrointestinal symptoms. These outcomes were compared in both groups.

The protocol for this research project was approved by a suitably constituted ethics committee of the institution (Committee of Ichinomiyanishi Hospital, Approval No. 2016071), and it conformed to the provisions of the Declaration of Helsinki (as revised in 2013). Informed consent was obtained from all participants, and this trial was registered with the University Hospital Medical Information Network (UMIN no. 000025006).

### Statistical analysis

If the length of the study term was <7 days, all measurements were used for analysis. The values of the primary end‐points were compared directly between the two groups. Interval data were expressed as the mean ± standard deviation, and categorical data as percentages. Statistical analysis was carried out using the Mann–Whitney test for the differences between the two groups; the χ^2^‐test was used to compare categorical variables. A value of *P* < 0.05 was considered statistically significant. All statistical analyses were carried out using EZR (Saitama Medical Center, Jichi Medical University, Saitama, Japan).

## Results

Overall, 58 patients with type 2 diabetes (41 medical and 17 surgical patients) consented to the study; four patients were excluded from further analysis, because they were transferred to the ICU or received systemic corticosteroid therapy, and one patient withdrew consent before starting the study. Thus, in both the BP and the BP + DU groups, 27 patients were included in the final analysis. The clinical characteristics of the study participants are shown in Table 2. There were no significant differences among groups in mean age, sex, body mass index, eGFR, duration of diabetes, admission glycated hemoglobin and BG levels, type of treatment before admission or the number of patients by primary admission diagnosis.

**Table 2:** Clinical characteristics of study patients

	Glargine	Dulaglutide + glargine	*P*‐value
No. patients	27	27	
Age (years)	70.1 ± 14	70.9 ± 13	0.822
Male, *n* (%)	18 (67)	15 (56)	0.5766
BMI (kg/m^2)^	24.6 ± 5.7	25.1 ± 6.7	0.904
Duration (years)	10.1 ± 8.8	8.7 ± 9.4	0.488
HbA1c (%)	8.0 ± 1.9	8.2 ± 1.7	0.401
Diet alone	7 (26)	10 (37)	0.558
Oral agents	18 (67)	11 (41)	0.101
Insulin + oral agents	2 (7)	6 (22)	0.25
Diagnosis			
Cancer, *n* (%)	2 (7)	0 (0)	0.491
Cardiovascular, *n* (%)	9 (33)	11 (41)	0.778
Head and neck, *n* (%)	2 (7)	1 (4)	1
Infection, *n* (%)	5 (19)	7 (26)	0.743
Orthopedic, *n* (%)	1 (4)	1 (4)	1
Plastics, *n* (%)	4 (15)	2 (7)	0.665
Renal, *n* (%)	2 (7)	4 (15)	0.665
Pulmonary, *n* (%)	3 (11)	1 (4)	0.603
Other, *n* (%)	4 (15)	4 (15)	1

Data are mean ± standard deviation or *n* (%). BMI, body mass index; HbA1c, glycated hemoglobin.

The main study outcomes are shown in Table 3. The primary outcome %hIGR was significantly higher in the BP + DU group than in the BP group (44% vs 56%, *P* < 0.001), whereas the frequency of BG <70 mg/dL and BG >240 mg/dL was significantly lower in the BP + DU group (2.3% vs 0.4%, 21% vs 8%, respectively; both *P* < 0.001). The secondary outcomes in the BP and BP + DU groups – mean BG (183 ± 29 vs 162 ± 30 mg/dL, *P* < 0.05), standard deviation (62.5 ± 21 vs 45.0 ± 14, *P* < 0.01), coefficient of variation (0.34 ± 0.09 vs 0.27 ± 0.05, *P* < 0.01) and total regular insulin (5.4 ± 2.5 vs 3.6 ± 2.8 U, *P* < 0.05) – were also significantly lower in the BP + DU group. The frequency of gastrointestinal symptoms was not different between the two groups (6% vs 11%, *P* = 0.241). The BP group experienced treatment failure, but no treatment failure occurred in the BP + DU group (3 vs 0%, *P* = 0.235).

**Table 3:** Main study outcomes

	BP group	BP + DU group	*P*‐value
	*n* = 27	*n* = 27	
Frequency of measuring BG levels for each BG range, *n* (%)			
BG <70	21 (2.3)	4 (0.4)	<0.001
BG 71–99	63 (7)	99 (10)	0.019
BG 100–180 (%hIGR)	399 (44)	545 (56)	<0.001
BG 181–240	232 (26)	244 (25)	0.748
BG >240	184 (21)	82 (8)	<0.001
Tges profiles, mg/dL (mean ± SD)			
Fasting	131 ± 38	127 ± 40	0.254
After breakfast	244 ± 61	196 ± 54	0.004
Before lunch	216 ± 65	173 ± 56	<0.001
After lunch	195 ± 62	182 ± 54	0.041
Before dinner	135 ± 47	143 ± 44	0.232
After dinner	203 ± 62	177 **±** 54	<0.001
Mean BG after first day	183 ± 29	162 ± 30	0.014
Glucose variability (mean ± SD)			
Individual glucose SD during study	62.5 ± 21	45.0 ± 14	0.001
Individual glucose CV during study	0.34 ± 0.09	0.27 ± 0.05	0.002
Hypoglycemic event, *n* (%)			
Patients <70 mg/dL	9 (33)	3 (11)	0.099
Patients <60 mg/dL	6 (22)	1 (3.7)	0.105
Patients <40 mg/dL	1 (3.7)	0 (0)	1.000
Insulin therapy, U/day (mean ± SD)			
Total insulin	17.1 ± 6.6	16.1 ± 6.4	0.762
Total glargine insulin	12.0 ± 5.7	12.6 ± 4.8	0.550
Total regular insulin	5.4 ± 2.5	3.6 ± 2.8	0.017
Others, *n* (%)			
Treatment failures	3 (1.1)	0 (0)	0.235
Gastrointestinal symptoms	6 (22)	11 (41)	0.241

Blood glucose (BG) measurements in the basal‐plus (BP) therapy group *n* = 974; BG measurements in the BP + dulaglutide (DU) therapy group *n* = 899. %hIGR, percentage of blood glucose measurements within the ideal glucose range for hospitalized patients among all blood glucose measurements CV, coefficient of variation; hIGR, ideal glucose range for hospitalized patients; SD, standard deviation.

**Figure 1:**
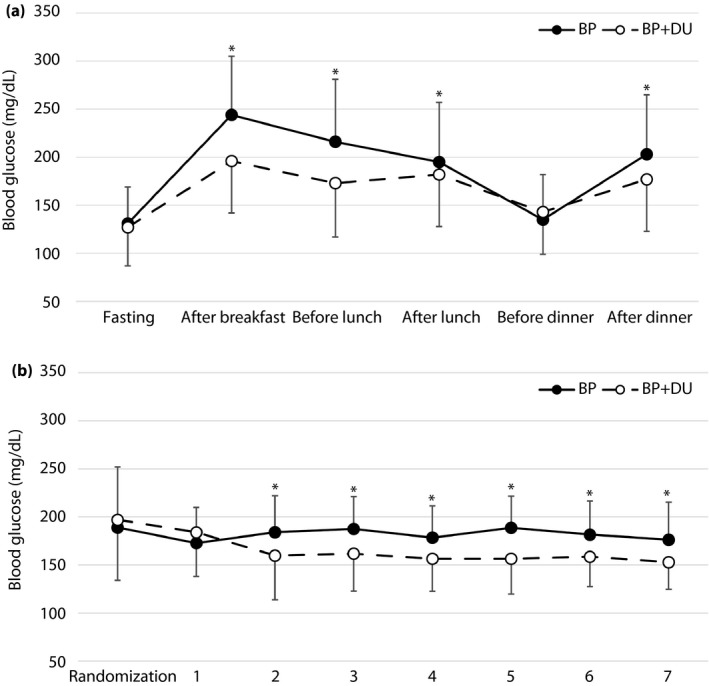
Differences in glycemic control among hospitalized patients with type 2 diabetes mellitus treated with basal‐plus (BP) therapy and BP + dulaglutide (DU) therapy. (a) Mean blood glucose concentrations before and after each meal. (b) Mean daily blood glucose levels during the study.

The mean BG concentrations measured before and after each meal in the two groups are shown in Figure 1a. There were no differences in FBG (131 ± 38 vs 127 ± 40 mg/dL, *P* = 0.25) and BG before dinner (135 ± 47 vs 143 ± 44 mg/dL, *P* = 0.23), whereas BG after breakfast (244 ± 61 vs 196 ± 54 mg/dL, *P* < 0.01), before lunch (216 ± 65 vs 173 ± 56 mg/dL, *P* < 0.001) and after dinner (203 ± 62 vs 177 ± 54 mg/dL, *P* < 0.001) were significantly lower in the BP + DU than in the BP group. Mean daily BG levels in the BP + DU group were also significantly lower than in the BP group from day 2 onwards (Figure 1b).

## Discussion

For patients living with type 2 diabetes, treatment with BP + DU increased the percentage of BG measurements that were in the ideal range, decreased the frequency of hyperglycemia and reduced the frequency of hypoglycemia compared with BP therapy alone. In addition, BP + DU therapy was able to reduce glycemic variability and the insulin correction dose.

BP therapy consists of a daily dose of basal insulin plus corrective doses with rapid‐acting or regular insulin given on a sliding scale. BP therapy is not inferior to a standard (BBT) therapy with insulin^21^, and is the preferred treatment for patients with poor oral intake or those who cannot accept medication orally^12^. Acutely ill patients with diabetes might not consume regular meals. Therefore, BP therapy is often selected in the initial stages of their treatment. The results of the present study show that BP + DU therapy can achieve superior glycemic control with reduced frequency of hyperglycemia and hypoglycemia compared with BP therapy alone. For these reasons, combined BP + DU therapy for non‐critical hospitalized patients might be useful in the critical initial stage of acute illness.

In the present study, patients in both groups received basal insulin therapy. Therefore, the results for FBG were similar in both groups, and there were no differences in the dose of basal insulin and total doses of insulin between the two groups. The only difference between the two groups was the administration of DU. Hence, differences observed in glycemic control between the two groups can be attributed to this distinction. Differences in glycemic control between the two groups were mainly observed in postprandial BG, suggesting that DU's mechanism of insulin secretion and glucagon suppression during hyperglycemia contributed to the lowering of postprandial BG. The frequency of hypoglycemia in the BP + DU therapy group did not increase, probably because of the glucose‐dependent effect of GLP‐1RA^18^. Taken together, these findings suggest that BP + DU therapy increased the likelihood of maintaining the ideal BG range, and suppressing GV and the need for additional insulin doses.

The benefits of incretin therapy in improving glycemic control in hospitalized patients have been previously reviewed^23^. The frequency of the target BG range has been previously reported to be significantly higher, and GV was significantly narrower in the exenatide group when comparing continuously intravenous GLP‐1RA (exenatide) therapy with insulin monotherapy^24^. The present results were in line with those findings, as we also observed a GV stabilizing effect with GLP‐1RA. Previous studies^17^, ^25^, ^26^ that compared the incretin‐related drug, dipeptidyl peptidase‐4 inhibitor, alone or combined with basal insulin to BBT in non‐ICU hospitalized patients, did not show any difference in the mean BG level. In those studies, which have combined an incretin‐related drug with basal insulin, the percentages of BG levels within a control range (70–140 mg/dL) to all BGs measured in each study were between 30.7–43%, mean BG was between 146.9–171 mg/dL and hypoglycemic events (BG < 70 mg/dL) occurred in 1–9% of patients. In the present study, BG was within a range of 100–180 mg/dL in 56% of our participants, the mean BG was 162 mg/dL and the percentage of patients with hypoglycemic events (BG <70 mg/dL) was 11%. Although these studies, including the present study, were evaluated with different numerical scales, all results of studies that combined an incretin‐related drug with basal insulin showed either non‐inferiority or superiority against the control group. Furthermore, another advantage of the treatment method in the present study was the reduced need for additional insulin. In view of these facts, we believe that the incretin‐related drugs, GLP‐1RAs and dipeptidyl peptidase‐4 inhibitors, can reduce GV. Hence, combining basal insulin therapy with these drugs might be a favorable combination to stabilize GV.

Basal–bolus insulin therapy is recommended as insulin therapy for non‐ICU hospitalized patients with diabetes, although this treatment method might increase the risk of hypoglycemia compared with SSI therapy^27^. Therefore, caution should be exercised when BBT is administered, and patients should be rigorously monitored for any signs of hypoglycemia. We observed that BD + DU therapy in the present study reduced the mean daily BG with low risk of hypoglycemia. We believe that DU plus conventional insulin therapy might be the ideal treatment method for insulin therapy for non‐ICU hospitalized patients with diabetes. In a recent outpatient study, combination therapy of basal insulin and a GLP‐1RA reduced hypoglycemia and suppressed GV compared with other treatment regimens, such as BBT^28^. Regardless of inpatient or outpatient treatment, combination therapy with basal insulin plus GLP‐1RA is highly promising in compensating for the disadvantage of insulin therapy alone.

GLP‐1RAs have several extrapancreatic effects; particularly, protective effects on the cardiovascular system. The administration of GLP‐1RA has been shown to: (i) counterbalance the deleterious effects of hyperglycemia or hypoglycemia on endothelial dysfunction, oxidative stress, and inflammation; (ii) reduce the risk factors for cardiovascular disease (systolic/diastolic blood pressure, triglycerides, low‐density lipoprotein cholesterol, high‐sensitivity C‐reactive protein, B‐type natriuretic peptide, inflammatory cytokines and oxidative stress); and (iii) improve cardiovascular and endothelial function^29^, ^30^. Furthermore, in preclinical studies, GLP‐1RA reduced myocardial infarct size^29^, ^30^.

The Liraglutide Effect and Action in Diabetes: Evaluation of Cardiovascular Outcome Results (LEADER) trial recently found significantly reduced cardiovascular‐related mortality and a lower risk of severe hypoglycemia with administration of a long‐acting GLP‐1RA (liraglutide), compared with non‐incretin therapies^31^. Regarding the likelihood of cardiovascular events after administration of DU, the recently carried out Researching Cardiovascular Events With a Weekly Incretin in Diabetes (REWIND) trial is currently being analyzed^32^. Therefore, the use of long‐acting GLP‐1RA in non‐critical hospitalized patients also has a potential for inhibiting cardiovascular events.

Gastrointestinal symptoms are also important extrapancreatic effects of GLP‐1RAs. Several GLP‐1RAs are currently available for treatment of patients with type 2 diabetes. Based on their pharmacokinetic/pharmacodynamic profiles, these drugs are classified as short‐acting GLP‐1RAs or long‐acting GLP‐1RAs^33^. The short‐acting type has a stronger delaying effect on gastric emptying compared with the long‐acting type; thus, gastrointestinal symptoms are stronger with the short‐acting type than with the long‐acting type. Therefore, in the present study, we considered that a long‐acting GLP‐1RA with fewer gastrointestinal symptoms^34^ and a longer incretin effect would be appropriate as supplementary treatment to insulin therapy for hospitalized patients in the acute phase of illness. DU, a long‐acting GLP‐1RA, is administered once weekly by injection. DU acts at a relatively early stage, and as a result, the hypoglycemic effect can be expected relatively early according to a study using continuous glucose monitoring in Japanese patients^35^. The present data also showed that treatment with BP + DU therapy improved the mean daily BG after the second day of therapy.

Furthermore, DU maintains its GLP‐1RA action for approximately 1 week. Therefore, DU has fewer daily fluctuations of the GLP‐1RA concentration than once‐daily liraglutide^36^. DU was used at a low dose of 0.75 mg in the present study, which we considered sufficient for an antihyperglycemic effect^37^, and this low dose is associated with a lower percentage of dose‐dependent gastrointestinal symptoms compared with high‐dose DU^38^. Nevertheless, gastrointestinal symptoms are a non‐negligible problem in using GLP‐1RA during hospitalization. Although there was no significant difference between the two groups in gastrointestinal symptoms in the present study, it should be avoided in patients with gastrointestinal disease and pre‐existing gastrointestinal symptoms. From the present analysis, we found no relationship between the presence or absence of gastrointestinal symptoms and the degree of glycemic control, but it is necessary to further investigate the effects of gastrointestinal symptoms on outcomes, such as hospital length of stay or food intake.

The present study, confirmed the superiority of BG control with BP + DU therapy compared with BP therapy, suggesting there is an indication for the prescription of GLP‐1RA to control BG in hospitalized patients.

Several limitations of the present study need to be mentioned. The first week of any acute disease is thought to have an important influence on the course of the disease later; however, prognosis and length of hospitalization could not be evaluated because of the short‐term nature of the study. Additionally, this was a pilot study; the sample size was small and the patients were recruited from only one hospital. Therefore, general administration of GLP‐1RA in hospitalized patients should be undertaken cautiously. It would be desirable to carry out a high‐quality investigation based on multicenter trials with a long‐term focus. Further study is required to examine the comparison with the gold standard insulin therapy, BBT, and to evaluate the cost‐effectiveness of GLP‐1RA, which is more expensive than insulin.

## Disclosure

The authors declare no conflict of interest.
